# Dioxins and Cardiovascular Disease Mortality

**DOI:** 10.1289/ehp.11579

**Published:** 2008-07-22

**Authors:** Olivier Humblet, Linda Birnbaum, Eric Rimm, Murray A. Mittleman, Russ Hauser

**Affiliations:** 1 Department of Environmental Health, Harvard School of Public Health, Boston, Massachusetts, USA; 2 Office of Research and Development, U.S. Environmental Protection Agency, Research Triangle Park, North Carolina, USA; 3 Department of Epidemiology, Harvard School of Public Health, Boston, Massachusetts, USA; 4 Department of Nutrition, Harvard School of Public Health, Boston, Massachusetts, USA; 5 Cardiovascular Epidemiology Research Unit, Beth Israel Deaconess Medical Center, Boston, Massachusetts, USA

**Keywords:** cardiovascular disease, dioxin, epidemiology, healthy worker effect, herbicides, ischemic heart disease, mortality, occupational exposure, PCB, TCDD

## Abstract

**Objective:**

In this systematic review we evaluated the evidence on the association between dioxin exposure and cardiovascular disease (CVD) mortality in humans.

**Data sources and extraction:**

We conducted a PubMed search in December 2007 and considered all English-language epidemiologic studies and their citations regarding dioxin exposure and CVD mortality. To focus on dioxins, we excluded cohorts that were either primarily exposed to polychlorinated biphenyls or from the leather and perfume industries, which include other cardiotoxic coexposures.

**Data synthesis:**

We included results from 12 cohorts in the review. Ten cohorts were occupationally exposed. We divided analyses according to two well-recognized criteria of epidemiologic study quality: the accuracy of the exposure assessment, and whether the exposed population was compared with an internal or an external (e.g., general population) reference group. Analyses using internal comparisons with accurate exposure assessments are the highest quality because they minimize both exposure misclassification and confounding due to workers being healthier than the general population (“healthy worker effect”). The studies in the highest-quality group found consistent and significant dose-related increases in ischemic heart disease (IHD) mortality and more modest associations with all-CVD mortality. Their primary limitation was a lack of adjustment for potential confounding by the major risk factors for CVD.

**Conclusions:**

The results of this systematic review suggest that dioxin exposure is associated with mortality from both IHD and all CVD, although more strongly with the former. However, it is not possible to determine the potential bias, if any, from confounding by other risk factors for CVD.

Dioxins, a class of environmental pollutants resulting from the production and combustion of chlorinated compounds, have been shown to cause cardiovascular toxicity in animals (Dalton et al. 2001; Jokinen et al. 2003; Kopf et al. 2007; Lind et al. 2004). Although a number of epidemiologic studies have examined the association of dioxin exposure with cardiovascular disease (CVD) morbidity or mortality, we found no published systematic reviews on this topic, possibly because dioxin epidemiology research has focused primarily on the association with various cancers [International Agency for Research on Cancer (IARC) 1997; National Academy of Sciences 2007]. In this review we focus on CVD mortality. Given the large worldwide burden of CVD, the potential role of dioxin exposure as a preventable risk factor could be of substantial public health and clinical interest, especially in the context of recent reports of elevated environmental dioxin levels in China (Leung et al. 2007; Li et al. 2007, 2008) and ubiquitous low levels worldwide (Schecter et al. 2006).

## Definition of “dioxin.”

The term “dioxin” refers to a diverse group of structurally related, environmentally persistent chemicals that exert toxic effects through a common pathway mediated by the aryl hydrocarbon receptor (Van den Berg et al. 1998, 2006). Dioxins include several types of polyhalogenated aromatic hydrocarbons: polychlorinated dibenzofurans (PCDFs); some types of poly-chlorinated biphenyls (PCBs); and polychlorinated dibenzo-*p*-dioxins (PCDDs), including 2,3,7,8-tetrachlorodibenzo-*p*-dioxin (TCDD), the most potent member of this class of chemicals. Although “dioxin” is also sometimes used to refer to TCDD alone, in this review we use the broader definition.

## Animal and laboratory evidence

Although studies have demonstrated that the fetal mammalian heart is a sensitive target of TCDD-induced teratogenicity (Thackaberry et al. 2005), only in the past few years have toxicologic studies demonstrated cardiovascular effects after exposure to dioxins in adult rats and mice. These effects did not seem to occur as a result of overt toxicity. Chronic exposure of rats to either TCDD or PCB-126, the most potent of the dioxin-like PCBs, led to a dose-dependent increased incidence of degenerative cardiovascular lesions, including cardiomyopathy and chronic active arteritis (Jokinen et al. 2003). PCB-126 also increased heart weight, serum cholesterol levels, and blood pressure in rats (Lind et al. 2004). Adult mice exposed subchronically to TCDD developed increased blood pressure and heart weight, as well as elevated markers of oxidative stress (Kopf et al., in press). Increased blood pressure and triglyceride levels were also observed after an acute high dose of TCDD in mice (Dalton et al. 2001). *ApoE*−/− mice exposed to subchronic doses of TCDD also developed earlier and more severe atherosclerotic lesions (Dalton et al. 2001). Some of these changes may be due to altered gene expression, inflammation, and oxidative stress (Arzuaga et al. 2007; Lund et al. 2005), whereas others may relate to direct affects on cardiomyocytes involving dioxin perturbation of key calcium signaling pathways leading to abnormal depolarization (Xie et al. 2006). Recent evidence that TCDD causes mitochondrial dysfunction in cell culture (Biswas et al. 2008) may suggest an additional mechanism for the effect of dioxin on the cardiovascular system. Such molecular, physiologic, and morphologic effects in rodent models all provide biological plausibility to the association observed in epidemiologic studies between exposure to dioxins and CVD mortality.

## Methods

### Search strategy and selection criteria

**Figure 1:**
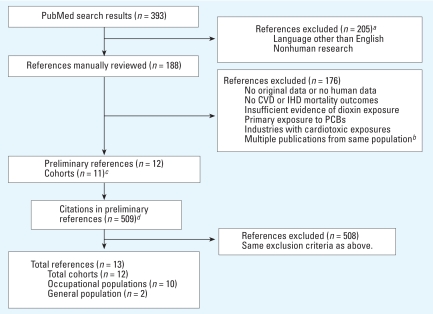
Flow diagram of study selection process using PubMed. ***a***Using the “limits” feature in PubMed. ***b***The only exception is described in “Methods.” ***c***The relevant results from the Hamburg study were divided into two publications. ***d***These 509 references include replicates of articles cited in multiple publications.

We found articles by searching PubMed (National Library of Medicine 2007) in December 2007 using the keyword “mortality” in combination with each of the following: dioxin, TCDD, PCDD, PCDF, phenoxy, chlorophenoxy, chlorophenol, and trichlorophenol. From this initial list, we selected studies that reported original epidemiologic data on mortality, in English, from either all cases of CVD, or ischemic heart disease (IHD). Other subtypes of CVD mortality (e.g., hypertension) were inconsistently reported; therefore, we did not examine them in this review. Figure 1 illustrates the study selection process.

We excluded studies with insufficient evidence of exposure to dioxin (e.g., ecologic studies, studies of Vietnam veterans not involved in herbicide spraying, or studies of pesticide applicators without information on which pesticides were used and whether dioxin contamination was possible). We also excluded studies whose primary exposure was to PCBs. Only 12 of 209 PCBs have dioxin-like activity, so exposure to non–dioxin-like PCBs would complicate the interpretation of any association of total PCBs with CVD.

Although dioxin exposure may occur during leather tanning and processing (Mikoczy et al. 1994) and in flavor and fragrance production (Thomas 1987), we excluded studies of cohorts from these industries due to the multiple cardiotoxic coexposures involved, including methylmercury, arsenic, and xylene.

If several follow-up studies had been published for a cohort, we included only the most recent results. By necessity, we made an exception when applying this rule to the IARC multicenter cohort, which updated and pooled the results of 36 individual cohorts (Vena et al. 1998). We did not include the earlier publications from these cohorts, as our protocol specified. The difficulty arose when four cohorts that were included in the IARC multicenter study subsequently published additional results: either additional follow-up time (Flesch-Janys et al. 1995; Steenland et al. 1999; ‘t Mannetje et al. 2005), or identical follow-up time but with more detailed exposure assessments (Hooiveld et al. 1998). Because the new results from these studies provided additional information, but the older results could not be dissociated from the summary relative risks (RRs) of the IARC study, we had no alternative but to effectively include these four studies twice; once as components of the IARC results, and then again as updated individual publications. Because these study results are therefore not independent, we did not attempt to quantitatively combine the RRs in a meta-analysis.

## Results

### Description of included studies

Eleven cohorts (Asp et al. 1994; Bertazzi et al. 2001; Flesch-Janys et al. 1995, 1998; Hertzman et al. 1997; Hooiveld et al. 1998; Ketchum and Michalek 2005; Ott and Zober 1996; Steenland et al. 1999; ‘t Mannetje et al. 2005; Tsai et al. 2007; Vena et al. 1998) remained after we applied the exclusion criteria to the 393 initial PubMed search results (Figure 1). We identified one additional cohort (Dalager and Kang 1997) by searching the citations, for a total of 12 included cohorts. Ten involved occupational or military exposure to dioxins, whereas the other two were environmentally exposed (Bertazzi et al. 2001; Tsai et al. 2007). One study was a large, multicenter cohort study (Vena et al. 1998) conducted by IARC, which examined noncancer mortality from 36 occupational cohorts and 26,976 workers potentially exposed to TCDD or other highly chlorinated dioxins. Of the 11 other cohorts, 4 were also part of the IARC study (Flesch-Janys et al. 1995; Hooiveld et al. 1998; Steenland et al. 1999; ‘t Mannetje et al. 2005), but we still included them separately in this review because they subsequently published additional follow-up or more detailed analyses.

Although quantitative dioxin exposure profiles for each cohort were not available because limited biological measurements were performed, broad exposure categorizations are possible. The workers in the 10 occupational cohorts were primarily exposed to PCDDs through the production and/or application of phenoxy acid herbicides and chlorophenols (Vena et al. 1998), with some possible PCDF exposure, as well (Becher et al. 1996). Of the remaining two studies, the Italian Seveso population (Bertazzi et al. 2001) was acutely exposed to high levels of environmental TCDD contamination after an industrial accident, whereas the Taiwanese Yucheng population (Tsai et al. 2007) was acutely exposed to a mixture of PCDFs and PCBs from ingesting contaminated rice oil. The latter study was included in spite of the PCB coexposures, because the toxic effects have been suggested to correlate more closely with exposure to PCDFs (Ryan et al. 1990).

### Criteria of study design quality

We grouped study results according to two well-recognized criteria of study design quality. The first criterion was whether mortality among the exposed participants was compared with an internal or an external reference group. External comparisons used standardized mortality ratios (SMRs) to compare the number of deaths observed in the exposed group (e.g., dioxin-exposed factory workers) with the number of deaths expected in the general population, standardized for age and sex. The primary limitation of external comparisons is that whenever the exposed group is an employed population, associations between exposure and CVD mortality will be biased downward (generally below the null) because workers are healthier on average than the general population (McMichael 1976) (“healthy worker effect”). Internal comparison studies instead contrast the highest-exposed groups with the lowest-exposed group in the study population, which minimizes the healthy worker effect bias if the cohort is occupational, as well as potential confounding by other factors that do not vary within the study population. For these reasons, internal comparisons are of higher quality than external comparisons.

The second criterion we used to evaluate studies in this review was exposure assessment quality. Many studies had little information on exposure level and therefore considered all members of the exposed group to be equally exposed. Because of these crude exposure estimates, the exposed groups may have contained individuals with low or no actual exposure. This nondifferential misclassification would tend to dilute any associations between exposure and CVD mortality, biasing the associations downward. In contrast, some studies were able to conduct more detailed exposure assessments, using exposure measures that varied in complexity from dichotomous yes/no exposure among workers (IARC), to septiles of cumulative exposure [National Institute for Occupational Safety and Health (NIOSH)]. Additional details on these studies’ personal exposure assessments can be found in the original articles (Bertazzi et al. 2001; Flesch-Janys et al. 1995; Hooiveld et al. 1998; Ketchum and Michalek 2005; Ott and Zober 1996; Steenland et al. 1999; Vena et al. 1998).

### Crude exposure assessments, with either internal or external comparisons

Table 1 presents the results of analyses using the crudest exposure assessments (i.e., only exposed vs. unexposed), with either internal or external comparisons. Most external comparison studies in this group found no elevated SMRs for either IHD or all CVD. This included the Seveso (Bertazzi et al. 2001) and Yucheng (Tsai et al. 2007) studies, even though no healthy worker effect bias was present because the exposed were part of the general population. Of the two internal comparison studies, the Ranch Hand study (Ketchum and Michalek 2005) showed a modestly elevated RR for all CVD (RR = 1.3; *p* = 0.07), whereas the Army Chemical Corps study (Dalager and Kang 1997) did not show an increased risk (RR = 1.06; 0.62–1.82).

### Detailed exposure assessments, with external comparisons

Four studies conducted external comparisons but stratified their SMRs by detailed exposure level (Bertazzi et al. 2001; Ott and Zober 1996; Steenland et al. 1999; Vena et al. 1998). Because external comparisons were used in these studies, healthy worker effect bias may be present in the three studies in which the exposed group had occupational exposure (Ott and Zober 1996; Steenland et al. 1999; Vena et al. 1998), but not in the fourth study (Seveso), in which the exposed group was environmentally exposed (Bertazzi et al. 2001). These results are not shown separately because three of the four studies also included internal comparisons (Table 2) and found similar results.

In the Seveso study, Bertazzi et al. (2001) calculated the SMRs in the highest and second-highest exposure zones. Exposure in this study was ecologically defined by proximity to the site of an industrial accident. The IHD SMRs [95% confidence intervals (CIs)] were 0.8 (0.4–1.5) and 1.0 (0.8–1.2), respectively, and were similar for all CVD. Although no trend was seen between the two highest-exposed zones at the 20-year follow-up, a significantly elevated RR for all circulatory disease was seen among the men in the highest-exposed zone, but only within 10 years of the accident (Consonni D, personal communication; data not shown), leading the authors to hypothesize that psychosocial stress was the cause (Bertazzi et al. 2001).

The IHD SMRs (95% CIs) for the IARC and NIOSH studies from lowest to highest exposure using the same categories as shown in Table 2, were 0.85 (0.77–0.94) and 0.97 (0.90–1.04) for IARC (Vena et al. 1998) and the SMRs were 0.93, 1.00, 1.05, 0.97, 1.10, 1.20, and 1.28 for NIOSH (Steenland et al. 1999) (no 95% CIs were shown; *p*-trend = 0.14). In the BASF study (Ott and Zober 1996), the IHD SMRs corresponding to the exposure categories of < 0.1, 0.1–0.99, and ≥ 1 μg/kg TCDD body weight were 0.9 (0.3–1.8), 0.7 (0.2–1.7), and 0.6 (0.2–1.3), respectively. All of these results are qualitatively similar to the internal comparison results for each study.

### Detailed exposure assessments, with internal comparisons

**Figure 2:**
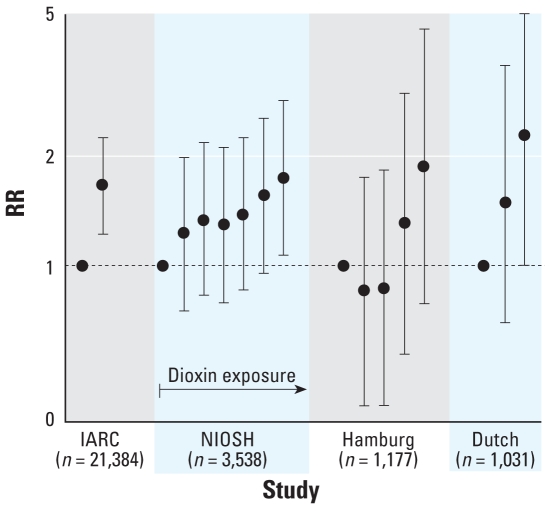
RRs (95% CIs) for IHD mortality from internal comparisons, by quantiles of dioxin exposure within each study. The exposure categories are not necessarily equivalent across studies (Table 2). The IARC study (Vena et al. 1998) includes the Dutch study (Hooiveld et al. 1998) and earlier versions of the NIOSH (Steenland et al. 1999) and Hamburg (Flesch-Janys et al. 1995) studies.

**Figure 3:**
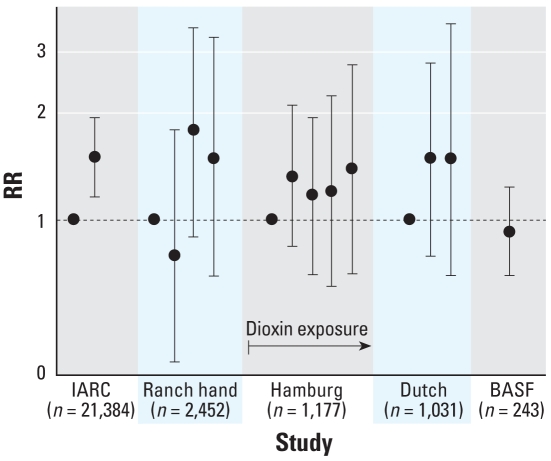
RRs (95% CIs) for all-CVD mortality from internal comparisons, by quantiles of dioxin exposure within each study. The exposure categories are not necessarily equivalent across studies (Table 2). The IARC study (Vena et al. 1998) includes the Dutch study (Hooiveld et al. 1998) and an earlier version of the Hamburg study (Flesch-Janys et al. 1995). The BASF study (Ott and Zober 1996) presented the RR only for a 1-μg/kg increase in estimated TCDD dose.

Table 2 presents the results that we considered to be the highest quality according to our criteria, because the studies used both internal comparisons and more detailed exposure assessments. The data from Table 2 are shown graphically in Figure 2 (IHD) and Figure 3 (all CVD). Dose-related increases in IHD mortality were seen in all four studies reporting this outcome (Table 2, Figure 2). *p*-Values for trend were calculated in the NIOSH (Steenland et al. 1999) and Hamburg (Flesch-Janys et al. 1995) studies and were *p* = 0.05 and *p* = 0.03, respectively. The RR in the highest exposure group was significantly elevated in the IARC (Vena et al. 1998), NIOSH (Steenland et al. 1999), and Dutch (Hooiveld et al. 1998) studies.

For all-CVD mortality, the dose-related increases in mortality were less clear than for IHD (Table 2, Figure 3). *p*-Values for trend given for the Ranch Hand (Ketchum and Michalek 2005) and Hamburg (Flesch-Janys et al. 1995) studies were *p* = 0.07 and *p* = 0.05, respectively. The RR for all-CVD mortality in the highest exposure group was significantly elevated in the IARC study (Vena et al. 1998), but not in the others. The BASF study (Ott and Zober 1996) found no association of dioxin with all CVD.

A major concern in all the reviewed studies was potential confounding by the major risk factors for CVD (e.g., diet, smoking, physical activity). If these risk factors were strongly associated with dioxin exposure, they could confound the association between dioxins and CVD, biasing it either upward or downward. Of the studies in Table 2, only the Ranch Hand (Ketchum and Michalek 2005) and BASF (Ott and Zober 1996) studies adjusted for possible confounding by some of the major risk factors for CVD [smoking and family history of heart disease in Ranch Hand; smoking and body mass index (BMI) in BASF]. The Ranch Hand study found moderately elevated adjusted RRs for all CVD (*p* for trend = 0.07). The BASF study found no elevated adjusted RRs for all CVD. Neither study examined IHD, and neither study presented crude RRs, which otherwise could have been compared with the adjusted RRs to assess the strength of confounding.

The Hamburg study (Flesch-Janys et al. 1995) reported stronger trends using estimated total I-TEQ [toxic equivalencies of each dioxin relative to TCDD (North Atlantic Treaty Organization, Committee on the Challenges of Modern Society 1988)] than using only estimated TCDD concentration (data not shown). The TEQ results, which represent the cumulative potency of the multiple dioxin congeners, are considered more biologically relevant than using TCDD alone. The Hamburg study was the only study to use both measures.

## Discussion

The present review synthesizes the epidemiologic studies of dioxin and CVD mortality and advances our understanding by considering in detail the studies according to their quality. Studies using external comparisons and crude exposure estimates found no association between dioxin exposure and increased risk of IHD or all-CVD mortality, but this may be due to healthy worker effect bias (McMichael 1976; Monson 1986; Weed et al. 1987) and nondifferential misclassification of exposure. In contrast, the higher-quality studies using internal comparisons and detailed exposure assessments found a consistent association between dioxin exposure and increased risk of IHD mortality, and a relatively weaker association between dioxin exposure and risk of all-CVD mortality. However, only two of these studies adjusted for possible confounding by some of the major risk factors for CVD. Additionally, it is not possible to determine from the published data whether the association between dioxin exposure and all CVD would persist if we excluded IHD cases.

The Seveso study (Bertazzi et al. 2001) was the only large study to report no exposure–response increase in CVD mortality, despite stratification of the exposed into low and high categories, both of which were compared with an external referent group. Although the reason for the different results is unknown, the Seveso study differs from the other studies in several important respects. The Seveso study population had a younger age structure, and the TCDD exposure due to an industrial accident was acute and was assessed ecologically. Whether these factors partially account for the different results is unknown.

### Limitations

The major limitation of these epidemiologic studies is the lack of adjustment for other major risk factors for CVD (including smoking, lack of physical activity, poor diet, and alcohol consumption). Only age was consistently adjusted for. The Ranch Hand (Ketchum and Michalek 2005) and BASF (Ott and Zober 1996) studies adjusted for some of the potential risk factors for CVD, but because the crude RRs were not shown, the magnitude of confounding could not be assessed. Several authors have suggested that potential confounders in occupational mortality studies are unlikely to be sufficiently highly correlated with both the exposure and the outcome to completely explain a positive association of the magnitude seen here (Blair et al. 2007; Kriebel et al. 2004; Siemiatycki et al. 1988). However, the possibility that the associations are affected by uncontrolled confounding cannot be excluded.

Some recent cross-sectional studies of dioxin exposure and CVD morbidity have found associations that persisted after more thorough adjustment for confounding (Ha et al. 2007; Kang et al. 2006; Kim et al. 2003). Ha et al. (2007) reported an association between PCDDs (but not PCDFs) and self-reported CVD in the 1999–2002 National Health and Nutrition Examination Study (NHANES). The adjusted odds ratios (ORs), by PCDD exposure quartile, in both sexes combined were 1.0 (reference category), 1.4, 1.7, and 1.9 (*p* for trend = 0.07) after adjustment for age, race, BMI, smoking, alcohol consumption, exercise, cholesterol, hypertension, and C-reactive protein. No crude ORs were shown. Kang et al. (2006), in a study of Army Chemical Corps Vietnam veterans, found that spraying phenoxy herbicides was associated with an OR (95% CI) for self-reported heart disease of 1.41 (1.06–1.89), adjusting for age, BMI, and regular smoking. However, other cross-sectional studies of cardiovascular morbidity have not found significant associations with dioxin exposure (Calvert et al. 1998), indicating that more research is needed. The human evidence relating dioxins to potential intermediate causes of CVD, such as serum lipids, is similarly mixed [U.S. Environmental Protection Agency (EPA) 2004].

The associations between dioxin exposure and increased risk of cardiovascular mortality were in study populations with dioxin levels substantially higher than U.S. general population levels (Ferriby et al. 2007; Patterson et al. 2008). However, Ha et al. (2007), using NHANES data, found an association between dioxin exposure and increasing prevalence of heart disease at U.S. general population levels. Several of the occupational studies that we reviewed presented the RRs among workers with low-to-moderate exposure levels that could be relevant to the general population. Although the RRs at low dioxin levels were elevated in several studies, the CIs were wide and the exposure categories were too broad to draw firm conclusions. More research is needed to characterize the CVD risk of low-level exposures, both in animal and in human studies.

The RRs from internal comparisons across the various studies are similar, even though the mean exposure levels differ. However, in each case the highest exposed category consisted of individuals with estimated exposures of comparable magnitude (Table 2). Therefore, the similar RRs seen in the highest-exposed group of each study may truly be due to similar exposures in this group. It is also worth noting that the CIs around each RR are very wide, indicating that, for example, the slightly larger RR seen in the highest exposure group of the Dutch study (Hooiveld et al. 1998) compared with the Hamburg study (Flesch-Janys et al. 1995) (RR = 2.3 vs. RR = 1.89) might only be the result of sampling variability because their 95% CIs overlap considerably (1.0–5.0 vs. 0.79–4.51). Additionally, the magnitude of an RR can be affected by uncontrolled confounding or by the background rate of CVD in the referent group, which are also likely to vary among studies.

Another weakness of the reviewed mortality studies is the difficulty of accurately retrospectively assessing personal exposure. However, this exposure misclassification is likely to be nondifferential with respect to CVD mortality, which would tend to decrease the observed associations in the highest exposure category (Blair et al. 2007; Dosemeci et al. 1990).

Most studies reported results only for TCDD, and not total TEQ, which is considered more biologically relevant. In addition, dioxin exposure was usually accompanied by coexposure to other contaminants, whose precise composition varied within occupational settings, and between occupational and environmental settings. It is a limitation of epidemiologic studies that separating out the effect of any one specific contaminant is difficult, especially given the possibility of synergism or antagonism. The primary occupational coexposure was to chlorophenols and their derivatives, but the available toxicologic studies of chlorophenols do not suggest that they have toxic effects on the cardiovascular system [Agency for Toxic Substances and Disease Registry (ATSDR) 1999].

Our findings do not directly address the risks of dioxin exposure for females because none of the internal comparison studies included substantial numbers of women. In a general population morbidity study, Ha et al. (2007) found very similar effects for men and women. However, another recent study found associations between dioxin exposure and mortality from both chronic rheumatoid heart disease and hypertension in women but not in men, whereas the association between dioxin exposure and mortality from all circulatory diseases was stronger in men (Consonni et al. 2008). Of the four animals studies we noted above, three used female animals (Dalton et al. 2001; Jokinen et al. 2003; Lind et al. 2004) and one used males (Kopf et al. 2007), and all observed cardiovascular effects. The limited evidence suggests that both sexes are sensitive to the cardiovascular effects of dioxins, although the possibility of the type and severity of effects differing by sex cannot be excluded.

The downward bias of the SMRs of occupational studies due to the healthy worker effect complicates their interpretation. This bias is illustrated by the substantially stronger associations found using internal comparisons than using SMRs in the IARC (Vena et al. 1998) and NIOSH (Steenland et al. 1999) studies, despite the same exposure categories being used for both analyses.

Although information on the mortality status of the participants was available at the time of the retrospective exposure assessment, this was unlikely to influence the estimation process in a way that might induce a relationship between the estimated dioxin exposure and CVD mortality because the *a priori* focus of these studies was cancer mortality.

These studies were also limited by their reliance on mortality and death certificate diagnoses. However, CVD mortality is likely to be diagnosed relatively accurately, and any errors would affect the precision but not the validity of the results. Virtually identical *International Classification of Diseases, Ninth Revision* (WHO 1978) codes were used to define IHD and all CVD in each study.

The IARC internal comparison results at least partially include the results from three of the other internal comparison studies (Flesch-Janys et al. 1995; Hooiveld et al. 1998; Steenland et al. 1999; described in “Methods” and in Figures 2 and 3). This raises the question of whether the associations reported in the IARC study might be solely due to its inclusion of these three studies. This seems unlikely, however, because the magnitude of the associations seen in the IARC study is similar to those seen in these three studies (Table 2), even though they represent only approximately 25% of the total number of participants included in IARC.

## Conclusions

The results of this systematic review suggest that dioxin exposure is associated with increased risk of mortality from both IHD and all CVD, although more strongly with the former. Although biological plausibility is provided by animal studies, uncontrolled confounding by other risk factors for CVD cannot be ruled out as a contributor to the association.

We hope our results will stimulate further evaluation of CVD incidence and mortality in dioxin-exposed cohorts, especially using internal comparisons with detailed exposure assessments, and careful control for confounding. Future studies in both animals and humans should assess whether cardiovascular effects are present at environmentally relevant doses. Of additional interest would be analysis of whether the association between dioxin exposure and all CVD persists when IHD cases are excluded, as well as a pooled or meta-analysis of the internal comparison results in order to obtain a dose–response curve for dioxin and CVD.

## Figures and Tables

**Table 1:** RRs (95% CIs) for mortality from IHD and all CVD from both internal and external comparisons with crude exposure assessments, in dioxin-exposed cohorts.

Comparison type/cohort	No.	IHD	All CVD	Reference
External				
Canadian sawmill^a^	23,829	NA	0.74 (0.71–0.76)	Hertzman et al. 1997
Seveso	6,745	1.00 (0.8–1.2)	1.00 (0.8–1.1)	Bertazzi et al. 2001
NIOSH	5,132	1.09 (1.00–1.20)	NA	Steenland et al. 1999
Finnish sprayers	1,909	0.94 (0.80–1.10)	NA	Asp et al. 1994
Yucheng	1,823	NA	1.00 (0.8–1.3)	Tsai et al. 2007
Hamburg	1,177	0.97 (0.77–1.22)	1.06 (0.90–1.24)	Flesch-Janys et al. 1998
New Zealand				
Production	813	1.04 (0.74–1.43)	0.96 (0.72–1.27)	‘t Mannetje et al. 2005
Sprayers	699	0.49 (0.31–0.75)	0.52 (0.36–0.73)	
Dutch	549	1.20 (0.8–1.6)	1.00 (0.8–1.4)	Hooiveld et al. 1998
BASF	243	0.70 (0.4–1.1)	0.80 (0.6–1.2)	Ott and Zober 1996
Internal				
Ranch Hand	20,340	NA	1.30 (1.0–1.6)	Ketchum and Michalek 2005
Army Chemical Corps	5,609	NA	1.06 (0.62–1.82)	Dalager and Kang 1997

NA, data not available.

a Using an alternate method of estimating person-time, the SMR for all CVD was 1.14 (1.10–1.18).

**Table 2:** RRs (95% CIs) for mortality from IHD and all CVD from internal comparisons, by dioxin exposure level.

Study	No.	IHD	All CVD
IARC (Vena al. 1998), TCDD/HCD exposure	21,384		
No	7,553	1.00 (—)	1.00 (—)
Yes	13,831	1.67 (1.23–2.26)	1.51 (1.17–1.96)
NIOSH^a^ (Steenland et al. 1999), cumulative exposure	3,538		
0 to < 19	505	1.00 (—)	NA
19 to < 139	505	1.23 (0.75–2.00)	NA
139 to < 581	505	1.34 (0.83–2.18)	NA
581 to < 1,650	505	1.30 (0.79–2.13)	NA
1,650 to < 5,740	505	1.39 (0.86–2.24)	NA
5,740 to < 20,200	505	1.57 (0.96–2.56)	NA
≥20,200	505	1.75 (1.07–2.87)	NA
		Trend *p*-value = 0.05	
Ranch Hand^b^ (Ketchum and Michalek 2005), dioxin exposure category (ppt lipid)	2,452		
Comparison	1,436	NA	1.00 (—)
Background	442	NA	0.80 (0.4–1.8)
Low (32.2–117.4)	287	NA	1.80 (0.9–3.5)
High (117.9–4221.9)	287	NA	1.50 (0.7–3.3)
			Trend *p*-value = 0.07
Hamburg^c^ (Flesch-Janys et al. 1995), total I-TEQ (ng/kg lipid)	1,177		
1.19–39.5	471	1.00 (—)	1.00 (—)
39.6–98.9	235	0.85 (0.41–1.75)	1.34 (0.85–2.13)
99.0–278.5	235	0.86 (0.41–1.83)	1.18 (0.71–1.95)
278.6–545.2	118	1.31 (0.57–3.00)	1.21 (0.66–2.25)
545.3–4361.9	118	1.89 (0.79–4.51)	1.40 (0.71–2.76)
		Trend *p*-value = 0.03	Trend *p*-value = 0.05
Dutch^d^ (Hooiveld et al. 1998), TCDD dose (ppt lipid)	1,031		
Low (7.1)	530	1.00 (—)	1.00 (—)
Medium (7.7–124.1)	259	1.50 (0.7–3.6)	1.50 (0.8–2.8)
High (124.2–7307.5)	242	2.30 (1.0–5.0)	1.50 (0.8–2.9)
BASF^e^ (Ott and Zober 1996), estimated TCDD dose	243		
1 μg/kg increase		NA	0.93 (0.70–1.24)

Abbreviations: HCD, higher chlorinated dioxins; NA, data not available.

a The number for each exposure group was estimated by dividing the sample into septiles, as done by Steenland et al. (1999). The cumulative exposure measure is a relative ranking; the units cannot be interpreted as a specific dose of dioxin.

b The serum dioxin levels were extrapolated back to the end of service in Vietnam. No extrapolated dioxin levels were presented for the comparison or background categories.

c The number for each exposure group was estimated by combining the two lowest quintiles and halving the highest quintile, as done by Flesch-Janys et al. (1995). The serum dioxin levels were extrapolated back to the end of occupational exposure.

d The serum dioxin levels were extrapolated back to the end of occupational exposure.

e This study presented the RR for all CVD only for a 1-μg/kg increase in estimated TCDD dose.
